# Identifying Multiple Potential Metabolic Cycles in Time-Series from Biolog Experiments

**DOI:** 10.1371/journal.pone.0162276

**Published:** 2016-09-27

**Authors:** Mikhail Shubin, Katharina Schaufler, Karsten Tedin, Minna Vehkala, Jukka Corander

**Affiliations:** 1 Department of Mathematics and Statistics, University of Helsinki, Helsinki, Finland; 2 Institute of Microbiology and Epizootics, Freie Univerität Berlin, Berlin, Germany; 3 Faculty of Medicine, University of Oslo, Oslo, Norway; Universite Paris-Sud, FRANCE

## Abstract

Biolog Phenotype Microarray (PM) is a technology allowing simultaneous screening of the metabolic behaviour of bacteria under a large number of different conditions. Bacteria may often undergo several cycles of metabolic activity during a Biolog experiment. We introduce a novel algorithm to identify these metabolic cycles in PM experimental data, thus increasing the potential of PM technology in microbiology. Our method is based on a statistical decomposition of the time-series measurements into a set of growth models. We show that the method is robust to measurement noise and captures accurately the biologically relevant signals from the data. Our implementation is made freely available as a part of an R package for PM data analysis and can be found at www.helsinki.fi/bsg/software/Biolog_Decomposition.

## Introduction

Biolog Phenotype Microarray (PM), not to be confused with RNA expression microarrays, is a commercially available 96-well format test system capable of multiple parallel testing of the bacterial growth responses to different nutrients and/or supplements [1]. The standard Biolog PM plates contain a variety of different substrates, such as carbon and nitrogen sources, heavy metals, antibiotics, etc. The substrates are pre-dispensed and dried, requiring only inoculation with bacteria and a buffer containing a dye (usually tetrazolium violet). Bacterial metabolism during growth leads to the irreversible reduction of the dye in the well with production of a purple colour which can be read as the change in absorbance over time [2]. The level of colouration is generally determined by a scanner at 15 minute intervals during the experiments, which are usually carried out over 48–72 hours depending on the studied bacterial species. The measurements of the colouration are recorded in arbitrary units.

**Fig 1:**
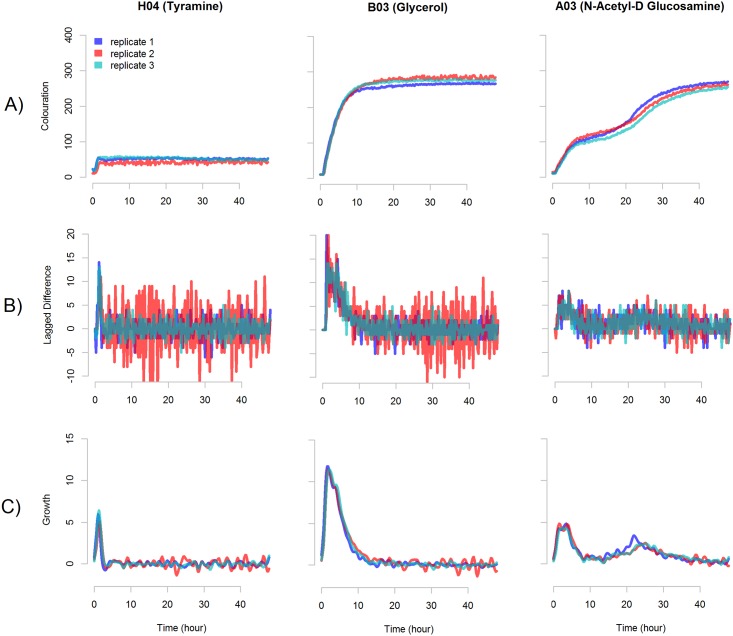
Kinetics of accumulation of the colour production may represent metabolic cycles in bacteria. Panel A—colour production of three replicates of the bacterial *E. coli* strain IMT17887 during growth on plate PM1 in the substrates H03 (Tyramine), B03 (Glycerol) and A03 (N-Acetyl-D Glucosamine). Panel B—lagged difference *L* of the colouration of these signals. Panels C—growth rate *S* (smoothed lagged difference of the colouration) of these signals. The smoothing coefficient is set to *b* = 0.5.

The levels of colouration measured in a single well during one experiment are here referred to as *signal*. Signals are normally consistent between experimental replicates (Fig 1A) and depend on bacterial adaptation to a substrate and experimental conditions. Preferred or utilisable substrates support active metabolism which is reflected by a rapid signal growth (Fig 1, substrates B03, A03). In contrast, toxic substrates inhibit bacterial growth or lead to cell death in which case only a small amount of colour is produced (Fig 1, substrate H04).

Bacteria may often undergo several cycles of metabolic activity, seen as differences in the rates of colour accumulation during growth (Fig 1, substrate A03). Multiple cycles may represent different metabolic pathways sequentially used by bacteria as they switch between nutrients, undergo depletion of substrates, or after excretion of end-products followed by re-utilisation. They may also represent different subpopulations in the growing cultures.

A number of methods have been used for analysing the Biolog metabolic signals and comparing the metabolic activity triggered by different substrates. The simplest approaches describe metabolic signals with a single summary statistic, e.g. maximum intensity reached or the area under the curve [3, 4]. Some methods split signals into growth or no-growth curves by using an arbitrary cut-off or comparison to a reference signal [5, 6]. However, describing a time-series with only a single summary statistics leads to a loss of information, and may introduce bias in the results [7]. If more than two samples are provided, the differences in summary statistics can be tested, e.g. by using t-test or ANOVA.

In addition to simple summary statistics, model-based methods are widely applied to Biolog data [7–10]. They are able to utilize more information by fitting growth models such as logistic, Gompertz and Richard, to the metabolic profiles. An R package opm is a widely used tool for reading in, processing and visualizing Biolog data [9]. It fits Gompertz’s and Richard’s models by using the grofit R package, and enables the comparison of the curves based on the 95% confidence intervals of the model parameter estimates. The most recent software by Gerstgrasser et al. [7] fits several models at once, and chooses the most suitable one utilizing Bayesian inference in parameter estimation and model selection. The signals are then compared against each other by defining maximum colour change, steepest slope and length of lag phase based on the fitted models. However, none of these growth models, or other methods [5, 6, 11–14] are able to capture more than one potential metabolic cycle at a time.

To address the problem, we propose an algorithm for identifying multiple potential metabolic cycles of bacteria by decomposing the PM well signal into multiple growth models. In addition, we propose a method for comparing signals with each other using summary statistics gained from the growth models. We show that the method is robust to measurement noise and captures accurately the biologically relevant information from the data, thus increasing the potential of PM technology in microbiology.

To illustrate the proposed algorithm, we use Biolog metabolic signals from three *E. coli* strains: IMT17887, PCV17887 and T17887. Three biological replicates per strain were tested on plate PM1. Strain number IMT (Institut für Mikrobiologie und Tierseuchen) 17887 was isolated from a horse with wound infection. It is an extended-spectrum beta-lactamase (ESBL)-producing *E. coli* of sequence type (ST) ST648. ESBL-plasmid extraction using a heat technique resulted in the ESBL-plasmid-“cured” variant PCV17887 [15]. Transformant T17887 contains the ESBL-plasmid, which was transferred into PCV17887 via electroporation. The data is provided in S1 File.

## Signal decomposition

The proposed algorithm is applied separately to each of the 96 wells on the PM plates. Here, the time-series *R* = (*R*_1_, *R*_2_ … *R*_*T*_) contain the raw signal, i.e. the sequence of *T* integers between 0 and 400 representing the measured intensity of the colouration in one particular well at several time points. In theory, as the production of the purple colour is irreducible, *R* would be an increasing sequence. In practice, *R* is subject to low-frequency observational noise and can show a decreasing pattern due to measurement errors.

As the metabolic activity is represented not by the absolute level of colouration but rather by its change due to growth of the cultures, we are not interested in the values in *R* as such, but the increments in the process. However, the lagged difference of the signal *L* = (*R*_1_, *R*_2_ − *R*_1_, *R*_3_ − *R*_2_,…,*R*_*T*_ − *R*_*T*−1_) is typically noisy (Fig 1B), which calls for a statistical approach to analyse the curves. To filter the noise the lagged difference is smoothed with a Gaussian kernel with a predefined smoothing coefficient *b* (Fig 1C). We define the target signal *S* as:

$$S_{t}=\frac{\sum_{\tau =1}^{T}L_{t}\;e^{-\frac{{\left(\tau -t\right)}^{2}}{b}}}{\sum_{\tau =1}^{T}e^{-\frac{{\left(\tau -t\right)}^{2}}{b}}}.$$ (1)

**Fig 2:**
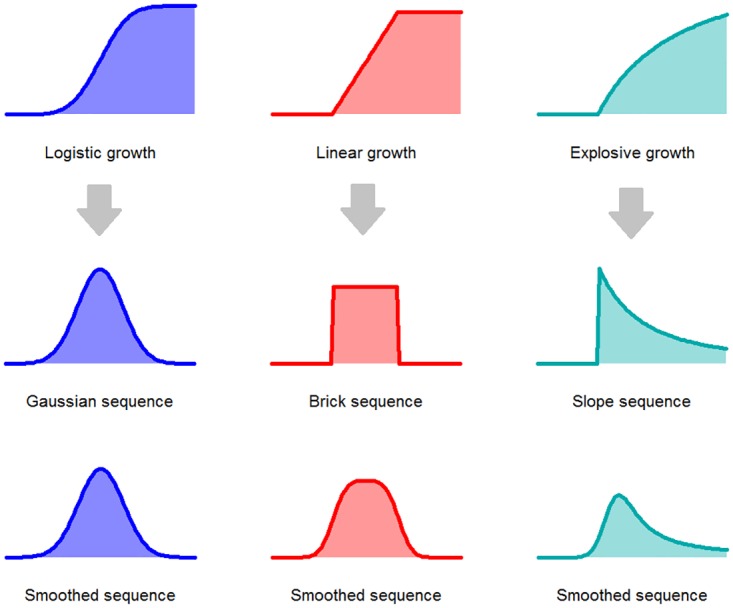
Basic components. The function used as components, smoothed functions and the type of growth represented by these components.

To identify metabolic cycles, the target signal *S* is approximated with the sum of *n* components $S\approx \sum_{i=1}^{n}C^{\left(i\right)}$, where each component $C^{\left(i\right)}=\left(C_{1}^{\left(i\right)},C_{2}^{\left(i\right)}\ldots C_{T}^{\left(i\right)}\right)$ represents one period of colour accumulation (e.g. potential metabolic cycle). We focus on three basic types of components based on the following growth models (Fig 2):

a Gaussian sequence:
  $$C_{t}=A\;e^{-\frac{{\left(\mu -t\right)}^{2}}{v}};$$
a brick sequence:
  $$C_{t}=\left\{\begin{array}{cc} 0 & t<t_{0}\\ A & t_{0}\leq t<t_{1}\\ 0 & t_{1}\leq t \end{array}\right.;$$
and a slope sequence:
  $$C_{t}=\left\{\begin{array}{cc} 0 & t<\mu \\ A\;\frac{{\left(\mu -t_{0}\right)}^{2}}{{\left(\mu -2t_{0}+t\right)}^{2}} & \mu \leq t \end{array}\right..$$

Here *A*, *μ*, *t*_0_, *t*_1_ and *v* are the component parameters. Each component is defined by exactly three parameters. To compensate for the smoothing, all sequences *C* are also processed by the Gaussian kernel with the same smoothing coefficient *b*.

Gaussian and brick sequences represent a logistic and a linear growth of a colouration respectively, while slope sequences represent a dynamics with initial growth slowing down with time (Fig 2). The various types of components are not intended to represent strictly different biological processes, but are used to increase the capability of matching the data patterns well.

**Fig 3:**
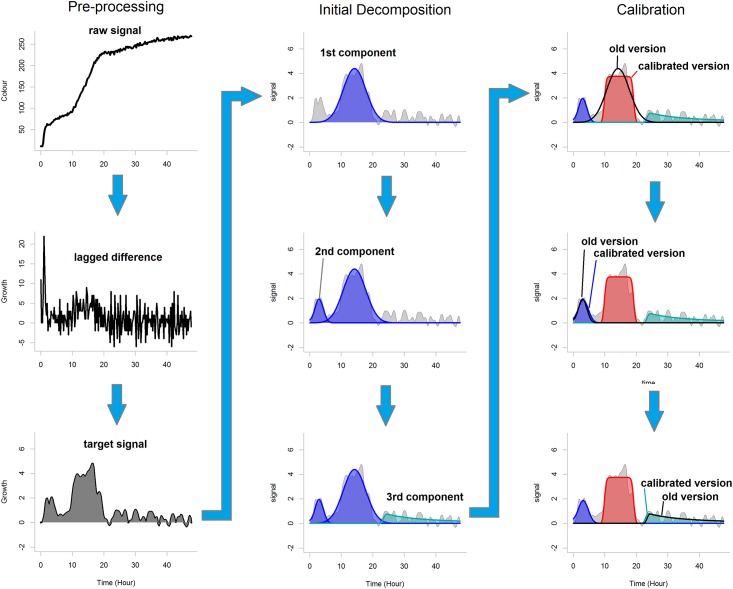
Decomposition of a Biolog signal. During the pre-processing raw signal is converted to the target signal. During the initial decomposition three components (putative cycles of metabolic activity) are revealed: two Gaussian sequences and a slope sequence. During the calibration these components are refined: the first component changes its type to a brick sequence, the second and the third are slightly adjusted.

**Fig 4:**
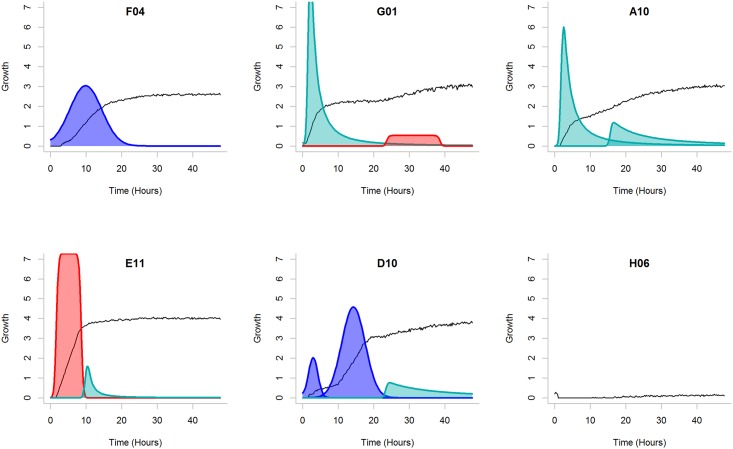
Example of the component decomposition. The signals generated by *E. coli* strain IMT17887 during growth on panel PM1. Substrates F04 (D-Threonine), G01 (Glycyl-L-Glutamic Acid), A10 (D-Trehalose), E11 (2-Deoxy Adenosine), D10 (Lactulose) and H06 (L-Lyxose) were analysed. The black lines show the analysed raw signal (not to scale). The different colour corresponds to the type of the components (turquoise for slope, red for brick and blue for Gaussian). One component was identified in F04, three in D10 and two in G01, A10 and E11. In H06 no growth is visible. The algorithm parameters were set as follows: blur strength *b* = 0.5, threshold *δ* = 20, correlation weight *γ* = 2.

Our decomposition algorithm consists of the following three steps: pre-processing converts the raw signal into the target signal; initial decomposition specifies the number of components required for optimal decomposition; calibration minimizes the distance between the components and the target signal and separates the components. To retain biological interpretability and prevent overfitting we set the constraint ∑*C*_*t*_ ≥ *δ* (where *δ* is a predefined threshold). Fig 3 shows an example of the algorithm processing a raw signal. Fig 4 shows the results of the decomposition for several PM signals.

**Pre-processing.** During the pre-processing step the target signal *S* is obtained by smoothing the lagged difference of the raw signal (see Eq 1). The smoothing is required to remove high-frequency observation noise.

**Initial decomposition.** During the initial decomposition step a crude decomposition is proposed using a greedy algorithm. The first component *C*^(1)^ is fitted to the whole signal *S*, the second component is fitted to the residuals *S* − *C*^(1)^, the third—to the residuals *S* − *C*^(1)^ − *C*^(2)^ and so on.

The fitting is done by optimizing *i*th component’s type and parameters to minimize squared error between the target signal and the proposed components:

$$C^{\left(i\right)}=\underset{C^{\left(i\right)}}{argmin}\sum_{t=1}^{T}{\left(S_{t}-\sum_{j=1}^{i}C_{t}^{\left(j\right)}\right)}^{2}$$

Optimization could in principle be done with any optimization algorithm capable of finding a global minimum in the restricted space of the parameters. In our implementation we use a grid method combined with the built-in R function optimize [16]. To choose the component type, we fit three different types separately and choose the one with a minimal squared error.

The iterations continue while fitted components satisfy the condition $\sum C_{t}^{\left(i\right)}\geq \delta$. When the first small component with $\sum C_{t}^{\left(i\right)}<\delta$ is encountered, the initial decomposition step is stopped and the *i* − 1 components are set as the initial decomposition. If the first proposed component is small $\sum C_{t}^{\left(1\right)}<\delta$ we conclude that no periods of colour accumulation have occurred (for example, no growth or the initial bacterial inoculum contained no live bacteria), the decomposition is not continued and a stale process is reported as a result.

**Calibration** Initial decomposition may be imprecise, as first components are obtained ignoring the later ones. If two or more components are found during the initial decomposition (*n* ≥ 1), the components are calibrated to achieve concordance between them. Components are calibrated sequentially: *C*^(1)^,*C*^(2)^,…*C*^(*n*)^,*C*^(1)^,*C*^(2)^… until a pre-determined condition is reached. It could be based on the number of iterations or a change in the distance between the components and the target signal.

When a component *C*^(*i*)^ is calibrated, its type and parameters are updated by minimizing the function:

$$\underset{1}{\underset{︸}{\overset{T}{\underset{t=1}{\sum }}{\left(S_{t}-\overset{i}{\underset{j=1}{\sum }}C_{t}^{\left(j\right)}\right)}^{2}}}+\underset{2}{\underset{︸}{\gamma \overset{T}{\underset{t=1}{\sum }}C_{t}^{\left(i\right)}\overset{n}{\underset{j=1;j\neq i}{\sum }}C_{t}^{\left(j\right)}}}$$

The first part of the function is the squared error between the proposed components and the target signal. The second part penalizes the correlation among the calibrated component and the rest of components. The latter is scaled with the correlation weight *γ*. The same optimization method as in the initial decomposition step is used.

Calibrating may change the type of a component. If a component ceases to satisfy the constraint $C_{t}^{\left(i\right)}\geq \delta$ after the calibration, it is removed and the rest of the components are re-calibrated.

## Using decomposition to compare signals

The summary statistics of the identified components could be used to measure a similarity between two signals and thus among replicates or different strains. We suggest three summary statistics:

max(C)

reflects the peak growth speed caused by the component;

$$size(C)=\sum_{t=1}^{T}C_{t}$$

estimates the size of the component (total gain in the colouration due to the component) and

$$center\left(C\right)=\sum_{t=1}^{T}tC_{t}/size\left(C\right)$$

measures the central time point in the component.

Using these summary statistics we can define a similarity measure between two components *A* and *B*:

$$sim\left(A,B\right)=\exp\left(-\frac{{\left(max\left(A\right)-max\left(B\right)\right)}^{2}}{2\delta_{\left(max\right)}^{2}}\right.\left.-\frac{{\left(size\left(A\right)-size\left(B\right)\right)}^{2}}{2\delta_{\left(size\right)}^{2}}-\frac{{\left(center\left(A\right)-center\left(B\right)\right)}^{2}}{2\delta_{\left(center\right)}^{2}}\right).$$

Here *δ*_(*max*)_, *δ*_(*size*)_ and *δ*_(*center*)_ are coefficients scaling the importance of the differences in summary statistics. *sim*(*A*, *B*) varies between 0 and 1 and does not depend on components’ types.

Finally we propose the following similarity measure for two decompositions A and B consisting of *m* and *n* components, respectively: $A=(A^{\left(1\right)},A^{\left(2\right)}\ldots A^{\left(m\right)})$ and $B=(B^{\left(1\right)},B^{\left(2\right)}\ldots B^{\left(n\right)})$. In case *m* ≠ *n*, assume A consist of more components (*m* ≥ *n*).

1. if *m* = *n* = 0 i.e. both signals are non-active, the similarity is set to 1;
2. if *n* = 0 and *m* > 0, i.e. if one signal is active and the other is non-active, the similarity is set to 0;
3. if *m*, *n* > 0: Compute the similarity as
  $$\prod_{i=1}^{n}sim\left(A^{\left(i\right)},B^{\left(i\right)}\right).$$
  Repeat the computations for each permutation of components inside the decomposition A, so that each component in A is compared to each component in B. Report the largest computed similarity.

This similarity metric varies between 0 and 1 and depends on summary statistics of the components. Since components in decompositions A and B may be in different order, and since decompositions may have incorrectly identified small false components, it is important to check all possible pairings between the decomposition and choose the best one. If decompositions have no components to compare, the metric is either set to 0 (one signal is active and one is non-active) or 1 (both are non-active).

**Fig 5:**
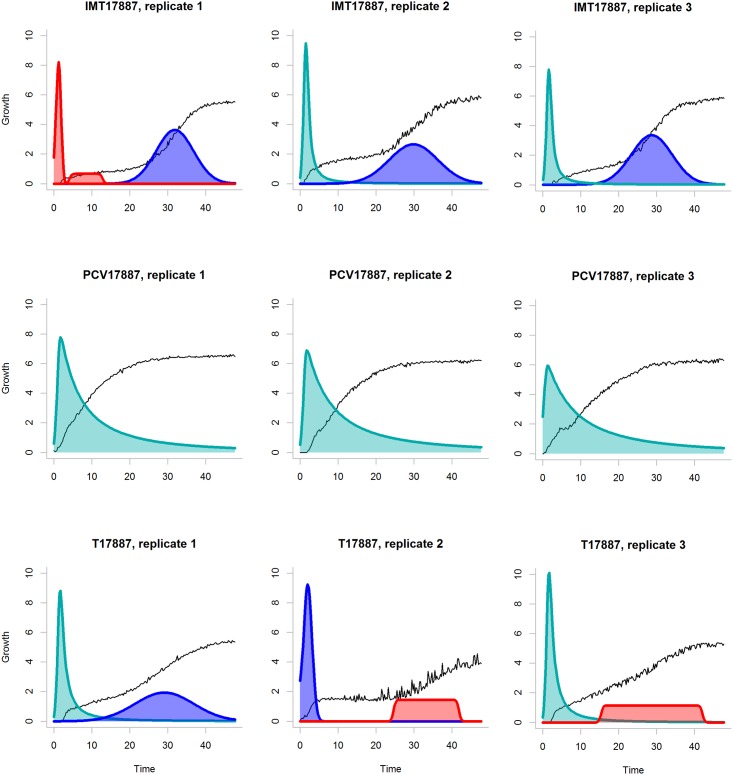
Example of the component decomposition. The signals of *E. coli* strains IMT17887, PCV17887 and T17887 (three replicates each) on plate PM1 and substrate A08 (L-Proline). The black lines show the analysed raw signal (not to scale). Colour corresponds to the type of the components. Raw signals and their decompositions are similar among replicates of the same strain. The algorithm parameters were set as following: blur strength *b* = 0.5, *δ* = 20, correlation weight *γ* = 2.

**Fig 6:**
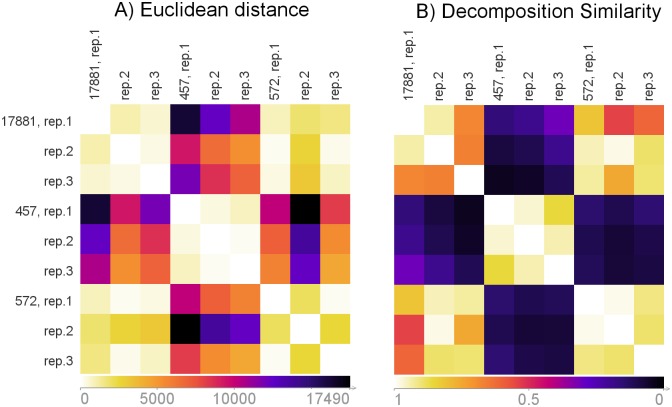
Example of the distance measure. The similarity was computed using the data and decomposition presented in Fig 5. Dark colour indicates less similar signals. Panel A—the Euclidean distance between the raw signals was used. Panel B—the similarity between signals is estimated as the similarity between their decompositions. To compute the distance we used *δ*_(*max*)_ = 3, *δ*_(*size*)_ = 100 and *δ*_(*center*)_ = 30.

Similarity between two decompositions can be used to cluster signals. Fig 5 shows the example of decomposition for signals of three *E. coli* strains (IMT17887, PCV17887 and T17887). The decompositions are consistent between experimental replications and vary between strains. Fig 6 shows the similarity measures computed for the same data based on the Euclidean distance (Panel A) and similarity between decompositions (Panel B). In the second case, the difference between the three *E. coli* strains is more pronounced. We recommend comparing the decompositions obtained with the same parameter values.

## Performance analysis

**Sensitivity test.** We tested the algorithm’s ability to identify the correct number and type of components and related summary statistics using a synthetic data set. First, we generated one component or a pair of negatively correlated components C*^(*i*)^ using randomly sampled parameters. The raw signal was constructed as a cumulative sum of components: $S_{t}^{*}=\sum_{\tau =1}^{t}C_{\tau }^{*}$. Non-normal noise was added to the raw signal:

$$S_{t}=max\left(0,S_{t}^{*}+Poisson\left(\lambda \right)-Poisson\left(\lambda \right)\right).$$

The non-normal noise was chosen to reflect the apparent non-normality of the Biolog observational noise. We then estimated the component (or components) *C*^(*i*)^ from *S*. We repeated the simulations 1000 times and measured a probability of correctly guessing the number of components *n*. If *n* was correct, we measured a probability identifying the component type and the mean absolute difference in the summary statistics *max*(*C*), *size*(*C*) and *center*(*C*). The results are shown in Table 1. The code used for testing is available at www.helsinki.fi/bsg/software/Biolog_Decomposition. We used *T* = 50 hours, blur strength *b* = 1, threshold *δ* = 20, correlation weight *γ* = 2 and the observation noise *λ* = 10.

**Table 1:** Sensitivity test.

Components	correctly identified *n*(%)	Type identified as			mean error		
		Gaussian(%)	brick(%)	slope(%)	max	size	center
one Gaussian	98	99	1	0	0.4	2.5	0.1
one brick	97	20	80	0	0.4	7	0.1
one slope	99	14	6	80	10	5	0.5
two Gaussian	71	66	27	7	1	66	2
two bricks	56	17	79	4	0.5	28	0.7
two slopes	74	15	32	53	12	23	1.7

Single components were almost always (in 97–99% cases) identified correctly. In 20% of the cases brick and slope components were misidentified because smoothing during the pre-processing step blurs the distinctions between the types. Narrow components are especially susceptible for this. The errors in the summary statistics were insignificant.

Identifying two components correctly was more challenging: the number of components was correctly identified in 56–74% of the cases. If the components were located close to each other, decomposition algorithm often mistook them as one or separated them in an incorrect position. The type of the components was correctly estimated in 53–79% of the cases. The errors in the summary statistics were larger in this setting as well.

**Fig 7:**
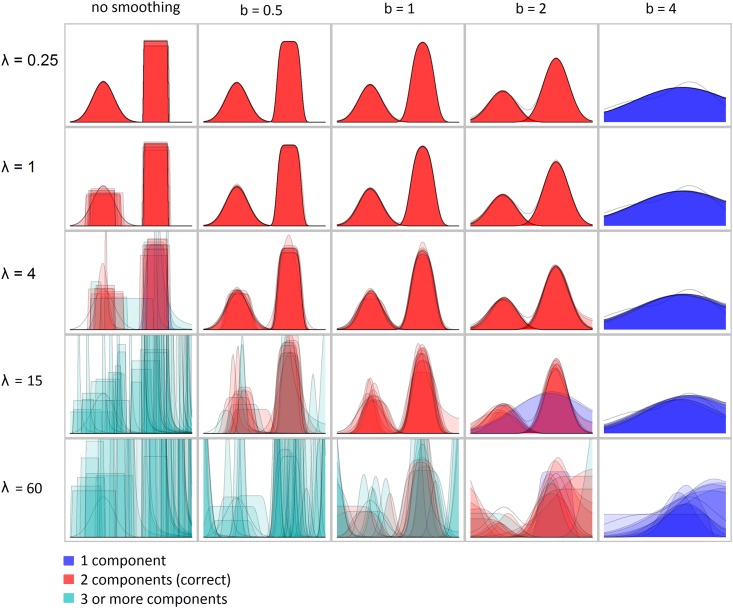
Decomposition of a single mock-up signal with different noise *λ* and smoothing coefficient *b*. Each subplot presents 10 decompositions superimposed one on the top of each other. The colour represents the number of components in the decomposition: blue for one, red for two, cyan for three or more. Gray lines show the correct decomposition smoothed with a corresponding coefficient *b*. Other parameters were fixed to the values *δ* = 20 and *γ* = 2.

**Robustness to parameter choice.** To assess the robustness of the decomposition algorithm to measurement errors and parameter choices with simulations, we applied the same protocol as in the sensitivity test above, but used fixed true components C* and varying values for the parameters *b*, *δ*, *γ* and observation noise *λ*. We tested several true component sets, five different values for all the parameters (*b* ∈ 0, 0.25, 0.5, 1, 1.5, *δ* ∈ 5, 10, 20, 30, 40, *γ* ∈ 0, 3, 10, 30, 100, *λ* ∈ 0.25, 1, 4, 16, 36) and 10 independent simulations for each combination of parameters. Fig 7 illustrates the results. The code used for testing is available at www.helsinki.fi/bsg/software/Biolog_Decomposition

Low values for the smoothing coefficient *b* lead to the identification of small false components while high values of *b* lead to the whole signal taken as one component (see Fig 7 for an example). The same applies to the component size threshold *δ*. Small values of correlation weight *γ* may lead to metabolic cycles being represented by several overlapping components, whereas large *γ* values may lead to metabolic cycles represented by several sequential components. Each data set may require an individual tuning of the parameter values for fully satisfactory results, which may be easily performed interactively by the user.

## Discussion

The proposed algorithm decomposes Biolog Phenotype Microarray data into potentially biologically meaningful components, i.e. components that could be interpreted directly as bacterial metabolic cycles and/or population changes. Identification of these components could be useful for further investigations, such as identifying sub-populations within bacterial cultures. Different signals (metabolic cycles) may arise after the initial death or growth stasis of a sub-population of bacteria followed by growth of a second sub-population. Also, metabolites generated during growth on the initial substrate might result in a second decomposition signal in a later phase of the experiment. Among the most promising future applications would be a direct link to concurrent RNA-sequencing data to detect different metabolic pathways.

The decomposition of growth kinetics and comparison of similarity among replicates and different strains is a meaningful tool for analysing the growth of different bacteria in a manner of high resolution, in contrast to methods only analysing the respiration kinetics data as end-point assays.

Performance analysis revealed that the presented method has a lowered sensitivity if there are several correlated components. Due to the observational errors, it is only possible to identify evident metabolic cycles. While the probability of correctly inferring the component type was low, the summary statistics were estimated accurately. Therefore any further analysis should rely on the summary statistics rather than on the component types.

The algorithm requires several pre-defined options to determine the sensitivity and level of smoothing. A user-specified tuning may be required to obtain an optimal fit for a particular data set. For some parameters, such as the component size threshold *δ* prior knowledge may also be used.

We have investigated different modifications of the basic algorithm. We tested to use an L1 norm instead of the L2 norm (Euclidean distance) to identify the components and using a sliding mean smoothing instead of a Gaussian kernel. Our analysis suggested (data not shown) that the presented version of the method provides the best sensitivity, specificity and robustness among the considered alternatives. We also considered including a fourth component type: a right half of a Gaussian bell (*C*_*t*_ = 0 for *t* < *μ*, $C_{t}=A\;e^{-\frac{{\left(\mu -t\right)}^{2}}{v}}$ for *t* ≥ *μ*). However, this half-Gaussian sequence was almost never observed in a sample data sets, as similar patterns are better described with a slope sequence.

The time-series generated by the Biolog PMs are inevitably subject to measurement noise. In addition to the signal-level noise (which is handled by the smoothing), there are plate-level biases, such that two plates with the same substrates in the same conditions may produce different amount of colouration. To handle the plate-level noise all time-series representative of the same array may be analysed concurrently and normalized *a priori*.

The presented algorithm does not require extensive computational resources. The runtime depends on the number of components identified and takes typically about 20 minutes to complete for a single plate in a standard single CPU desktop computing environment. The code is written in R and can be downloaded at www.helsinki.fi/bsg/software/Biolog_Decomposition. It is a part of a pipeline for analysing Biolog PM data [8] (www.helsinki.fi/bsg/software/R-Biolog) built upon the opm package [9].

## Supporting Information

S1 File Sample data. Biolog metabolic signals from *E. coli* IMT17887, PCV17887 and T17887 tested on plate PM1. Three biological replicates per strain.
(ZIP)

## References

[1] Biolog homepage;. Available from: http://www.biolog.com/.

[2] Khatri B, Fielder M, Jones G, Newell W, Abu-Oun M, Wheeler PR. High throughput phenotypic analysis of Mycobacterium tuberculosis and Mycobacterium bovis strains’ metabolism using biolog phenotype microarrays. PLoS ONE. 2013;8(1):e52673 10.1371/journal.pone.0052673 23326347 PMC3542357

[3] Bochner BR. New technologies to assess genotype-phenotype relationships. Nat Rev Genet. 2003;4(4):309–314. 10.1038/nrg1046 12671661

[4] Bochner BR. Global phenotypic characterization of bacteria. FEMS Microbiol Rev. 2009;33(1):191–205. 10.1111/j.1574-6976.2008.00149.x 19054113 PMC2704929

[5] Tohsato Y, Mori H. Phenotype profiling of single gene deletion mutants of E. coli using Biolog technology. Genome Inform. 2008;21:42–52. 19425146

[6] Tohsato Y, Baba T, Mazaki Y, Ito M, Wanner BL, Mori H. Environmental dependency of gene knockouts on phenotype microarray analysis in Escherichia coli. J Bioinform Comput Biol. 2010;8 Suppl 1:83–99. 10.1142/S021972001000521X 21155021

[7] Gerstgrasser M, Nicholls S, Stout M, Smart K, Powell C, Kypraios T, et al A Bayesian approach to analyzing phenotype microarray data enables estimation of microbial growth parameters. Journal of Bioinformatics and Computational Biology. 2016; p. 1650007 10.1142/S0219720016500074 26762475

[8] Vehkala M, Shubin M, Connor TR, Thomson NR, Corander J. Novel R pipeline for analyzing biolog phenotypic microarray data. PLoS ONE. 2015;10(3):e0118392 10.1371/journal.pone.0118392 25786143 PMC4365023

[9] Vaas LA, Sikorski J, Hofner B, Fiebig A, Buddruhs N, Klenk HP, et al opm: an R package for analysing OmniLog(R) phenotype microarray data. Bioinformatics. 2013;29(14):1823–1824. 10.1093/bioinformatics/btt291 23740744

[10] DeNittis M, Querol A, Zanoni B, Minati JL, Ambrosoli R. Possible use of Biolog methodology for monitoring yeast presence in alcoholic fermentation for wine-making. J Appl Microbiol. 2010;108(4):1199–1206. 10.1111/j.1365-2672.2009.04547.x 19796093

[11] Orro A, Cappelletti M, D’Ursi P, Milanesi L, Di Canito A, Zampolli J, et al Genome and Phenotype Microarray Analyses of Rhodococcus sp. BCP1 and Rhodococcus opacus R7: Genetic Determinants and Metabolic Abilities with Environmental Relevance. PLoS ONE. 2015;10(10):e0139467 10.1371/journal.pone.0139467 26426997 PMC4591350

[12] Blumenstein K, Macaya-Sanz D, Martin JA, Albrectsen BR, Witzell J. Phenotype MicroArrays as a complementary tool to next generation sequencing for characterization of tree endophytes. Front Microbiol. 2015;6:1033 10.3389/fmicb.2015.01033 26441951 PMC4585013

[13] Yan Q, Power KA, Cooney S, Fox E, Gopinath GR, Grim CJ, et al Complete genome sequence and phenotype microarray analysis of Cronobacter sakazakii SP291: a persistent isolate cultured from a powdered infant formula production facility. Front Microbiol. 2013;4:256 10.3389/fmicb.2013.00256 24032028 PMC3759002

[14] Scaria J, Suzuki H, Ptak CP, Chen JW, Zhu Y, Guo XK, et al Comparative genomic and phenomic analysis of Clostridium difficile and Clostridium sordellii, two related pathogens with differing host tissue preference. BMC Genomics. 2015;16:448 10.1186/s12864-015-1663-5 26059449 PMC4462011

[15] Schaufler K, Wieler LH, Semmler T, Ewers C, Guenther S. ESBL-plasmids carrying toxin-antitoxin systems can be “cured” of wild-type Escherichia coli using a heat technique. Gut Pathog. 2013;5(1):34 10.1186/1757-4749-5-34 24245987 PMC4177129

[16] Brent RP. Algorithms for Minimization Without Derivatives. Dover Books on Mathematics. Dover Publications; 1973. Available from: https://books.google.fi/books?id=6Ay2biHG-GEC.

